# Results of a Campaign for Motorcycle Helmets Advocacy in a City in Southwest of Iran; A Population-Based Intervention Study

**DOI:** 10.29252/beat-070410

**Published:** 2019-10

**Authors:** Ali Foroutan, Seyed Taghi Heydari, Mehran Karvar, Leila Mohammadi, Yaser Sarikhani, Maryam Akbari, Kamran Bagheri Lankarani

**Affiliations:** 1 *Shiraz Burn and Wound Healing Research Center, Shiraz University of Medical Sciences, Shiraz, Iran*; 2 *Health Policy Research Center, Institute of Health, Shiraz University of Medical Sciences, Shiraz, Iran*; 3 *Health Human Resources Research Center, School of Management and Information Sciences, Shiraz University of Medical Sciences, Shiraz, Iran*

**Keywords:** Motorcycle, Helmets, Advocacy, Interventions, Road traffic accident.

## Abstract

**Objective:**

We conducted a triple phase project for motorcycle helmets advocacy in Darab, a city in southwest Iran. The aim of this study was to evaluate the effect of the project on decreasing the hazards of motorcycle accidents.

**Methods:**

Using a questionnaire, data for ICU admission rates, hospital costs for patients who required ICU admission, rate of helmet usage, mortality and the duration of ICU care for patients admitted to Darab hospital due to motorcycle accidents in Winter 2015 (before conducting the project) and Winter 2016 (after conducting the project) were gathered and compared. This feature was also separately done for patients younger than 17 years.

**Results:**

The rate of wearing helmets increased significantly in winter 2016 (from 3.4 % to 33%). Also ICU admission rate due to head trauma was significantly decreased after the project was done (from 14.5 % to 4%). However, hospital costs for patients required ICU admission were increased in winter 2016. This increase, though not significant, seems to be due to an increase in health service expenses in the year 2016 as compared with the year 2015. The mortality rate was not significantly changed between the two mentioned years results. For patients younger than 17 years, no ICU admissions were needed in winter 2016.

**Conclusion:**

Even a short period of intervention can have positive effects on increasing the safety of motorcycle drivers.

## Introduction

Road Traffic Accidents (RTAs) are the third most common causes of death worldwide. It has been estimated that more than 1.2 million people are killed on roads due to RTAs annually [1]. Among different types of road injuries, motorcycle accidents are considered as the most fatal ones [2,3]. Studies have reported that RTAs occurs 9 times higher in riding motorcycles than the other types of vehicles [3]. In developing countries, the burden of RTAs is much higher than that of the developed world [4]. Although only 15% of all vehicles are motorcycles, 39% of all deaths caused by RTAs in Iran are due to motorcycle accidents [5,6].

The most common cause of death in motorcycle accidents is traumatic brain injury. In fact, in one third of the victims, brain is the sole organ injured [7]. Using helmets has been shown to be quite effective in preventing head injuries [8,9]. Based on a study, the use of a helmet reduces injuries by 69% and mortality by 42% [10]. Also, Intensive Care Unit (ICU) admission rates are significantly lower among victims who used a helmet. It has been shown that legislation for obligatory helmet wearing and also enforcement of the law can be very effective in increasing helmet usage and decreasing mortality rates [6,11,12]. Education is also considered as an effective strategy [13].

Darab is a city in south of Iran with about 201000 populations (based on results of the 2016 National Population and Housing Census from Statistical Center of Iran) and it has a warm and dry climate. Darab is unique for having more than 300 villages around it. Many people including high school students travel to Darab every day from villages for work or education. There is no public transport in this city. The main transporting method is using motorcycles. According to local police report, more than 100000 motorcycles are used for transportation in the town and suburbs. Based on reports from Darab Education Council, twenty-four high schools are available in Darab with roughly 1900 high school male students. In Darab, children start driving motorcycles as early as primary school ages. Although motorcycle helmet law has existed for more than 10 years in Iran, rate of wearing helmet is low in the country [14] which is even worse in small cities like Darab. Since transportation means between the villages are not sufficient, motorcycle seems to remain the main transportation method for several years to come. Thus, helmet use is vital for safety in cities that have the same structure as Darab.Zarrindasht, another city near Darab, has a population of 73000 people (based on results of the 2016 National Population and Housing Census from Statistical Center of Iran). Although Zarrindasht is smaller than Darab, it has the same structure as Darab regarding helmet and motorcycle use. Having the importance of preventing these types of accidents in mind, the authors conducted a 7-month project of helmet advocacy in this city which especially focused on children. The higher focus in children was due to the limitation of resources for intervention. The aim of the project was to improve the rate of helmet use an also to lower the risk of head trauma in motorcyclist in Darab. 

## Materials and Methods

*Setting*

The project duration was 7 months (from September 2015, until March 2016) and its effects were evaluated through the data gathered from Darab hospital in the last 3 months of the project (winter) from January to March 2016. The data were compared with the same months in the previous year and with patients from Zarrindasht city. The study protocol was approved by the institutional review board (IRB) and the medical ethics committee of Shiraz University of Medical Sciences, Shiraz, Iran. All the participants provided their informed written consents before inclusion in the study. 

*Before Interventions*

One year before Starting the project, the study team designed a questionnaire to assess the attitude of Darab teenagers toward using helmets. The questionnaire was filled by 396 school-aged male students in four high schools which were randomly selected. The questionnaire contained 20 questions about motorcycle driving experiences and use or non-use of helmets. Informed consents were taken before filling questionnaires. The followings are some examples of the questions:

Have you ever ridden a motorcycle?

How long have you been riding motorcycle?

Do you use motorcycle to attend your school?

*Interventions*

The project of advocacy and law enforcement contained 3 phases. The first phase was education and announcement for 1 month. Phase 2 was to give notifications and warnings to the riders, and phase 3 was the law enforcement. All of the phases were done as planned (Table 1).

*Phase 1*

The project was designed and prepared by the general surgeon of our team (first author). The surgeon arranged a meeting with the education correspondents, police officers, the governor, and the attorney general in September 2015. In the meeting, he clarified the purpose and aims of the project and asked for help of all authorities. Then, the study team started to educate high school students, traumatized patients in emergency department of Darab hospital, and clerks in governmental organizations about the dangers of motorcycling without a helmet and the importance of using standard helmets.

An amount of money was granted by Shiraz University of medical sciences for the purchase of helmets for students. Also, by order of the governor, all motorcycle sellers in the city were obligated to sell one helmet with each motorcycle. Moreover, it was decided that 20% of the money collected from motorcyclists’ penalties be allocated to the continuance of this project. A video clip for social media and four large billboards to be installed in crowded areas of the city were designed to announce the project and the importance of helmet use. Moreover, two local newspapers interviewed the surgeon by which he clarified the goals of the project.

The general surgeon with the help of a police officer presented a half hour lecture in all boy’s high schools. The lecture included topics around possible injuries, experiences of the surgeon about trauma mortality in the city, and the importance of helmet use for preventing head injuries and death. After purchasing 1700 helmets, they were distributed among the students of all high schools throughout the city within 3 weeks. Since bringing motorcycles into the school environment is prohibited, students park theirs on the street or in public parking spaces around the school. Thus, establishment of a motorcycle parking lot for students was discussed with school representatives for the purpose of monitoring the students’ safe use of motorcycles. Parents agreed to give informed consents by signing a letter, obligating motorcycle-riding students to wear helmets, otherwise, they would not be allowed to attend school. In each school, a teacher was made responsible to check fidelity to helmet rule among the students every day.

*Phase 2*

In this phase, the people were warned of the fines and financial penalties of riding a motorcycle without using helmet. Patients with RTAs were warned by the emergency room staff in Darab Hospital. Also, police officers and prehospital paramedics orally informed motorcyclists in busy pathways about the importance of helmet use and notified them about the plan beginning in the following month in which pressure increased for helmet law. A ticket of notifications was given to each individual after explaining the project to them. Six police officers and 5 paramedics cooperated for this phase voluntarily. This was done for 30 days and police estimated that nearly 2000 tickets were distributed.

*Phase 3*

In December 2015, police began enforcing the helmet law. This means that the police started to strongly restrict the riders without helmet in the streets taking their motorcycle into police station as a penalty for their breaking the law of having helmet. Paramedics and some hospital staff cooperated with police at random points inside the city. Also, some pathways at the entrance to the city were randomly monitored by police. All the governmental offices obligated their personnel to wear helmets while coming to work by motorcycle.

Educating the patient admitted due to motorcycle accidents was also continued during this phase.

**Table 1:** Phases of the population based campaign for motorcycle helmet advocacy

**Phase 1 (1 month)**	**Phase 2 (1month)**	**Phase 3 (3 months)**
1.Organizing the team for the project 2.Education of safe riding for high school student 3. Education of traumatized patients in emergency room 4. Grant allocation from the University penalty incomes 5. Distribution of inexpensive helmets among high school students. 6.Broadcasting the educational video clips and announcing the project in billboards and news paper 7. Establishment of high school parking and obligation of helmet use	1.Warning riders in streets 2. Ticket of notifications were distributed.	1.Taking the motorcycle of the disobedient riders 2. Education of disobedient in police station 3. obligation of personnel of governmental offices for helmet use 4.Continuing the obligation in high schools and emergency room

**Table 2:** Comparing victims of motorcycles in two successive years in two cities

	**Darab**			**Zarrindasht**		
**Year**	2015	2016	p	2015	2016	p
**Number of patients**	103	147	-	58	91	-
**Helmet use**	1 (1.0)	29 (19.7)	<0.001	0	0	-
**Mortality**	3 (2.9)	2 (1.4)	0.338	0 (0.0)	2 (2.2)	0.521^a^
**Motorcyclists ICU admission rates due to head trauma **	15 (14.6)	6 (4.1)	0.003	6 (10.3)	10 (11.0)	0.901^ a^
**Female patients**	6 (5.8)	7 (4.8)	0.709	8(13.8)	8 (8.8)	0.336^ a^
**Duration of ICU admission Mean ± SD**	5.27±6.22	6.00±6.93	0.815	5.17±4.88	7.80±7.29	0.448
**Costs for patients required ICU admission $ ± SD**	1932.43±1050.80	2473.53±239.22	0.467	2065.90±973.88	3173.19±2448.24	0.313

Numbers in parentheses are %; ^a^ fisher exact test

**Table 3:** Comparing paediatric patients in two successive years in the two cities

	**Darab**			**Zarrindasht**		
**Year**	2015	2016	p	2015	2016	p
**Number of patients**	29	35	-	14	26	-
**Helmet use**	0 (0)	11 (31.4)	0.001	0	0	-
**Mortality**	3 (10.3)	0 (0)	0.088	0	1 (3.8)	0.991^ a^
**Motorcyclists ICU admission rates due to head trauma **	6 (20.7)	0 (0)	0.006	4 (28.6)	3 (11.5)	0.214^ a^
**Female patients**	2 (6.9)	1 (2.9)	0.586	0 (0)	3 (11.5)	0.539
**Duration of ICU admission Mean ± SD**	9.0±8.63	-	-	4.0±2.45	4.67±3.51	0.777
**Costs for patients required ICU admission $ ± SD**	2548.42±1412.96	-	-	1575.31±576.43	2535.19±278.37	0.519

Numbers in parentheses are %; ^a^ fisher exact test

*Data Collection*

For the period between January 2015 and March 2016, using a questionnaire, the rate of helmet use among the patients admitted to the hospital due to motorcycle accidents, sex and age of them, the ICU admission rates among motorcyclist patients with head injuries, and the length of ICU stay, were calculated by the research team. Hospital expenses were collected from the hospital accountant. With permission from Darab Hospital’s manager, the same data for the same months in the year 2015 was also collected from patients’ profiles in the hospital.

*Statistics and Data Analysis*

In order to evaluate the effect of the interventions, we compared the results obtained in the winter when the interventions started with the results of the winter of the year before. Moreover, as it was mentioned before, Zarrindasht city has a very similar culture and trends toward motorcycle use as compared to those of Darab city. Since Darab hospital also covers the patients referring from Zarrindasht city, patients referred from Zarrindasht due to motorcycle accidents were considered as a control group. Therefore, data of these patients were compared with the data of the patients from Darab.

The analysis of data was conducted using statistical package for social sciences (SPSS Inc., Chicago, Illinois, USA) version 22. Mean and standard deviations are considered as descriptive statistics for continues variables and count and percent are used for categorical variable. Independent sample T test was done in order to compare costs and duration of ICU admission between two groups. Also, chi square test was performed to compare helmet use, motorcyclists’ ICU admission rates due to head trauma and sex between two groups. P value less than 0.05 was considered as the level of statistical significance.

## Results

*Before Interventions*

The questionnaire was filled by 396 high school students. The mean age of students was 16.2 years. Only two students reported that they had not ever ridden a motorcycle. Results showed that 257 students (64.9%; 95% CI: 62.5 to 67.3) rode motorcycle to go to school. The number of years by which the students experienced riding motorcycles ranged from 1 to 10 years and the average was 3 years. Only 7 (2.7%; 95% CI: 0.7 to 4.7) students claimed that they used helmet during motorcycle rides. The students were asked about their reasons for not using helmet. The question was open ended, the authors read the answers and categorized them in order to be reportable. The results showed that 123 students (31.1%; 95% CI: 28.8 to 33.4) reported feeling embarrassed by wearing a helmet and believed that using helmets is culturally unacceptable, 80 students (20.2%; 95% CI: 18.2 to 22.2) said that helmets were too expensive for them to buy, and 64 of them (16.1%; 95% CI: 14.3 to 18.1) claimed that it was hard to wear it in the boiling weather of Darab city. Other minor reasons included lacking ability to tolerate the heaviness of helmets, believing that using helmets is not necessary, thinking that wearing a helmet would be dangerous, and never having thought of using a helmet. Some students expressed shamefulness in wearing a helmet, saying: “People will make fun of us if we use a helmet” and “Our culture does not accept the use of a helmet “

*After Interventions*

With the cooperation of transportation police, a count was taken of motorcyclists wearing helmets on two major roads inside the city by direct observation for one week. This was done with the aid of three accredited observers who were responsible to observe the motorcyclists for 1 hour in the morning, one hour in the evening and one hour at night. Based on our survey, in 2015, about 7 (2.7%) of motorcyclists wore helmets; this figure increased to about one third (33%) by the winter of 2016. Since they were in 2 different years they might not be comparable, however both data were obtained in winter. 

Table 2 shows the data obtained from patients who admitted to Darab hospital due to motorcycle accidents based on the year of their admission and the city they came from (Darab or Zarrindasht). Totally, there were 161 and 238 patients (including patients both from Darab and Zarrindasht cities) who were admitted due to motorcycle accidents in winter of 2015 and 2016, respectively. In both years, most of the patients were men. 

Of those study patients from Darab, 29 (19.7%) patients wore helmets in winter 2016, while, in winter 2015, helmet usage was only one (1.0%). Mortality rate was three in winter 2015, and two in winter 2016, indicating no significant change. Furthermore, as compared with the results in winter 2015, there was a significant decrease in ICU admissions due to head trauma in winter 2016, which indicates a decrease in dangerous head injuries. Conversely, ICU admission rate in patients from Zarrindasht county where our project was absent, showed no significant difference in the two successive winters.

The mean duration of ICU admissions for patients from Darab was not significantly different between the 2 years (*p*=0.815). The financial burden of ICU admissions in both years were compared. The mean hospital costs for patients who required ICU admission in winter 2016 was higher than that of winter 2015. Although the mean of costs was higher in 2016, total costs of all patients (mean hospital costs of ICU admitted patients multiplied by their number) were much higher in 2015. These numbers do not include the reimbursements which insurance companies make for accident injuries. Nearly 90% of costs are paid by health insurance companies. Total and mean expenses in patients from Zarrindasht were higher in 2016 as compared with 2015.

*Teenagers*

Table 3 shows the results of the intervention among all teenagers in the two successive years. Since most of the preventive policies were aimed at high-school-aged children, the rates specifically in children aged 17 or younger were analysed. The results showed that 29 patients in winter 2015 and 35 patients in winter 2016 under the age of 17 years had motorcycle accidents. None of the victims in 2015 wore helmets; Whilst in 2016 helmet usage significantly increased in these patients. No mortality occurred in this age group in 2016, and there was a significant decrease in ICU admissions in that year as well. The same analysis was done for patients from Zarrindasht. None of the patients in either of the years wore helmets. There was no significant difference in ICU admission and mortality rates among patients under 17 years of age from Zarrindasht between winter 2015 and winter 2016. Comparison of proportions was used to evaluate changes in ICU admitted patients. A significant decrease in ICU admissions in Darab patients compared to the Zarrindasht patients was seen after the preventive intervention.

## Discussion

RTAs are among the leading causes of death worldwide. About 85% of all RTAs’ mortalities occur in developing countries [15]. More than 50% of mortalities are due to head injury [16]. The effectiveness of helmet use in preventing head injury is well documented [1,17,18] and many countries legislated the obligatory law of helmet use, even though many people argue that such a law may be a reduction of freedom [19]. Iran has legislated the use of helmets, however, enforcement of the law is perfunctory which reduces the effectiveness of it.

According to WHO reports [14], the rate of helmet use in Iran is about 35%, however, this rate is estimated for major cities. Smaller cities still have much lower rates of wearing helmets. Rate of wearing helmets in Darab city, according to local estimates, was about 3.4% before our project was applied. People may have some false beliefs against using helmets, for example: using helmets may increase the incidence of cervical spine injuries, decreases ability of the driver to hear alarming sounds, and limits the field of vision [6,7,20,21]. According to our study results, among Darab students, the most common cause of hesitance to wear a helmet is culture; they were ashamed to use helmets in the town. Educating students about the importance of wearing helmets seems to be very effective for helmet advocacy. Also, since many of them mentioned economic problems regarding buying helmets, providing financial supports such as subsidies for buying helmets seems to be a logical policy.

In 2016, there was a higher rate of helmet use. This increment was seen mainly in children since most of the advocacies and policies were related to the age group under 17 years old. In fact, no teenager from Darab was admitted to ICU due to head trauma caused by motorcycle accidents in winter 2016. Our result showed that after the project, the ICU admission rate and the total costs of ICU admitted patients for motorcycle injury in Darab decreased as compared to either the results in the previous year or results from the patients from Zarrindasht city. Among adult patients from Darab, the mean duration of ICU admission was almost the same in both years probably because none of the ICU admitted patients used helmets

Results showed that the mean hospital cost of patients requiring ICU admission was increased in winter 2016 as compared with that of winter 2015. This increase, though not significant, seems to be caused by an increase in health service expenses in 2016 due to economic inflations and also an increase in tariffs for medical services in the country. The higher hospital admission rates in winter 2016 were probably due to changes in insurance law that made it obligatory to admit patients if they were going to be observed in hospital for more than 6 hours. So far, many studies have addressed the benefits of using helmets in decreasing the burden of RTAs in motorcyclists. 

In a study in Taiwan on adult motorcyclists and bicyclists (n = 9321) it was concluded that use of motorcycle helmets provided protection to adult motorcyclists and decreased the mortality rates and the risk of head injuries [22]. These findings are consistent with the results of current study. In a systematic review including analysis of 60 U.S. studies, authors showed that implementing universal helmet laws are effective in increasing motorcycle helmet use and reducing deaths and injuries in motorcyclists of all ages, including young riders. Moreover, they concluded that repealing universal helmet laws decreased helmet use and increased deaths and injuries [23]. Results of a large study including 73,759 operators of motorcycles from eleven states in U.S. concluded that medical expenses and rates of head, facial, and brain injuries among motorcyclists were lower in states implementing universal law. Motorcyclists in partial law states suffered more from head, facial, and traumatic brain injuries. These differences support the effectiveness of universal helmet laws. They showed that wearing helmets was effective in reducing injury in both helmet law settings [24]. Patel P *et al*. conducted a retrospective analysis of 10,345 patients divided into 2 groups: those who used helmets (n = 6,250) and those who did not (n = 4,095). The group in which the patients were not wearing a helmet had greater risk of requiring ICU admission [25]. The results of current study are consistent with those of larger studies as indicated above. 

## Limitation

One of the problems we encountered was the limited enforcement that police is able to provide in a small town. As most of the residents in a small community know each other, social constraints hinder police officers from enforcing motorcycle and helmet-use laws. Also, stopping a motorcyclist is not as easy as arresting a 4 wheel vehicles because it has potential risk of injury especially at high speeds. The intervention reported herein was limited to 7 months, as boiling weather in spring and summer along with a local political election in spring prevented the project from being continued for a complete year. Furthermore, the money allocated for buying helmets, education and law enforcement ran out. Distributing low cost helmets in schools and governmental organizations is not easy. Finding those individuals who really need helmets and preventing the misuse and sale of the helmet in higher price should be considered in order to save resources. Even short period of intervention can have positive effect on increasing the safety of motorcycle drivers. Short term policies cannot be maintained for a long period.

## Conclusion

It is true that several factors might affect the variables measured in our study; however, since we compared the data from Darab with those from Zarrindasht, where the infrastructures, transportation means, authorities’ policies, socio-economic level of the families and many other factors are almost the same as Darab, we could conclude that the promising results we observed in Darab were generally due to the effects of our project. Findings of current study showed that helmet advocacy decreases health services costs for health insurance companies. Thus, it seems that health insurance companies should be strongly involved in the projects such as ours.

## Funding:

This study was funded by a grant from Shiraz University of Medical Sciences.

## Authors’ Declaration

The funder had no financial or proprietary interest in any material or method used in this study and had no role in study design, data collection and analysis, or preparation of the manuscript. 

## Conflict of Interest:

 The authors declare that there is no conflict of interests.
